# GAMBLING AND PSYCHOLOGICAL WELL-BEING AMONG OLDER CHINESE ADULTS IN THE US

**DOI:** 10.1093/geroni/igad104.2716

**Published:** 2023-12-21

**Authors:** Yuyang Zhu, Yiming Ma, Fengyan Tang, Yanping Jiang

**Affiliations:** Rutgers University, Piscataway, New Jersey, United States; Rutgers University, New Brunswick, New Jersey, United States; University of Pittsburgh, Pittsburgh, Pennsylvania, United States; Rutgers University, New Brunswick, New Jersey, United States

## Abstract

Gambling is a common but not harm-free social practice. Few studies have investigated the long-term impact of gambling activities on psychological well-being among racial/ethnic minority groups in the U.S. This study aimed to close this gap by examining the associations between gambling, problem gambling, and psychological well-being among older Chinese immigrants. We drew data from 2,811 (58% female) participants aged 60 years or older in the Population Study of Chinese Elderly in Chicago Study who completed the baseline assessment and at least one of the three follow-ups that were conducted every two years. Gambling participation was assessed using Modified South Oaks Gambling Screen and Problem Gambling Severity Index. The responses were used to categorize participants: never gambled (the reference group), participated in gambling, and problem gambling. Psychological well-being was assessed using Patient Health Questionnaire-9 and Hospital Anxiety and Depression Scale. Of all participants, 761 reported ever participating in gambling activities, and 52 reported problem gambling experiences. Participants who participated in gambling reported the lowest baseline scores of depression and anxiety compared to the other two groups. However, gambling participation predicted faster increases in depression (β=0.076, p=0.01) and anxiety (β=0.103, p=0.02) over time. Also, problem gambling was associated with a faster increase in anxiety (β=0.083, p=0.02). These results show a faster increase in psychological distress related to gambling participation over time. Culturally responsive interventions to reduce gambling activities and their impacts on psychological well-being are needed to promote health among this population.

